# Supplementary material for: Empirical methods for controlling false positives and estimating confidence in ChIP-Seq peaks
Source: BMC Bioinformatics. 2008 Dec 5;9:523. doi: 10.1186/1471-2105-9-523 (PMC2628906; doi:10.1186/1471-2105-9-523)
Supplement: Additional File 1 — A variety of html documents from the USeq web site detailing the available applications, their best usage, output file type descriptions, command line menus, etc. [file 1471-2105-9-523-S1.zip › USeqUserGuides/JavaDoc.zip!/JavaDoc/util/bio/parsers/gff/Gff3Feature.html]

Gff3Feature


|  |  |  |
| --- | --- | --- |
| **Overview** \| **Package** \| **Class** \| **Use** \| **Tree** \| **Deprecated** \| **Index** \| **Help** | |  |
| **PREV CLASS**   **NEXT CLASS** | **FRAMES**    **NO FRAMES**     **All Classes** |  |
| SUMMARY: NESTED \| FIELD \| CONSTR \| METHOD | DETAIL: FIELD \| CONSTR \| METHOD |  |


---


## util.bio.parsers.gff Class Gff3Feature

```
java.lang.Object
  util.bio.parsers.gff.Gff3Feature
```

**All Implemented Interfaces:**: java.lang.Cloneable, java.lang.Comparable

---

``` public class Gff3Feature extends java.lang.Object implements java.lang.Comparable, java.lang.Cloneable ```

A container for each of the values in a GFF3 line. See http://flybase.net/annot/gff3.html.
Does not look for multiple values in the attributes tag=value1,value2,value3 keeps value as a single String.

---

**Constructor Summary**

`Gff3Feature()`

`Gff3Feature(java.lang.String unParsedGff3Line)`
  
          Always check if the GFF3Feature is valid after instantiating with this constructor.


| **Method Summary** | |
| --- | --- |
| `java.lang.Object` | `clone()` |
| `int` | `compareTo(java.lang.Object obj)`             Sorts by the sortBy field so set it before sorting. |
| `java.lang.String` | `getAlias()`             Reserved Attribute. |
| `java.lang.String` | `getAttributes()` |
| `java.lang.String[]` | `getCustomAttributes()`             Any Attribute that is not a reserve attribute is grouped here. |
| `java.lang.String` | `getDbxref()`             Reserved Attribute. |
| `int` | `getEnd()` |
| `java.lang.String` | `getGap()`             Reserved Attribute. |
| `java.lang.String` | `getId()`             Reserved Attribute. |
| `java.lang.String` | `getName()`             Reserved Attribute. |
| `java.lang.String` | `getNote()`             Reserved Attribute. |
| `java.lang.String` | `getOntologyTerm()`             Reserved Attribute. |
| `java.lang.String` | `getParent()`             Reserved Attribute. |
| `int` | `getPhase()` |
| `double` | `getScore()` |
| `java.lang.String` | `getSeqId()` |
| `java.lang.String` | `getSortBy()` |
| `java.lang.String` | `getSource()` |
| `int` | `getStart()` |
| `java.lang.String` | `getStrand()` |
| `java.lang.String` | `getTarget()`             Reserved Attribute. |
| `java.lang.String` | `getType()` |
| `boolean` | `isEndSet()` |
| `boolean` | `isPhaseSet()` |
| `boolean` | `isScoreSet()` |
| `boolean` | `isStartSet()` |
| `boolean` | `isValid()`             Call to find out if the unparsed GFF line correctly parsed. |
| `static void` | `loadAttribute(java.lang.StringBuffer sb, java.lang.String reserveWord, java.lang.String value)` |
| `static void` | `loadField(java.lang.StringBuffer sb, java.lang.String field)` |
| `static void` | `saveGFF3TextFile(Gff3Feature[] g, java.io.File newGFF3File)`             Prints an array of GFF3Features as a plain text file |
| `void` | `setAlias(java.lang.String alias)` |
| `void` | `setCustomAttributes(java.lang.String[] customAttributes)` |
| `void` | `setDbxref(java.lang.String dbxref)` |
| `void` | `setEnd(int end)` |
| `void` | `setEndSet(boolean endSet)` |
| `void` | `setGap(java.lang.String gap)` |
| `void` | `setId(java.lang.String id)` |
| `void` | `setName(java.lang.String name)` |
| `void` | `setNote(java.lang.String note)` |
| `void` | `setOntologyTerm(java.lang.String ontologyTerm)` |
| `void` | `setParent(java.lang.String parent)` |
| `void` | `setPhase(int phase)` |
| `void` | `setPhaseSet(boolean phaseSet)` |
| `void` | `setScore(double score)` |
| `void` | `setScoreSet(boolean scoreSet)` |
| `void` | `setSeqId(java.lang.String seqId)` |
| `void` | `setSortBy(java.lang.String sortBy)` |
| `void` | `setSource(java.lang.String source)` |
| `void` | `setStart(int start)` |
| `void` | `setStartSet(boolean startSet)` |
| `void` | `setStrand(java.lang.String strand)` |
| `void` | `setTarget(java.lang.String target)`             Reserved Attribute. |
| `void` | `setType(java.lang.String type)` |
| `void` | `setValid(boolean valid)` |
| `java.lang.String` | `sgrBundle()`             Returns a collection of sgr lines representing the gff feature: 1) seqId startMin1 0 2) seqId start score 3) seqId end score 4) seqId endPlus1 0 Does not include a final return, thus println(gff.sgrBundle()). |
| `java.lang.String` | `toString()`             Generates a GFF3 line. |
| `java.lang.String` | `toStringNoAttributes()`             Generates a GFF3 line. |

**Methods inherited from class java.lang.Object**

`equals, getClass, hashCode, notify, notifyAll, wait, wait, wait`

**Constructor Detail**

### Gff3Feature

```
public Gff3Feature()
```

---


### Gff3Feature

```
public Gff3Feature(java.lang.String unParsedGff3Line)
```

:   Always check if the GFF3Feature is valid after instantiating with this constructor.
    Invalid cases arise from not having 9 tab delimited columns or having an attribute
    that does not split in two on '='. Escaped characters are OK. Will also fail if
    numbers could not be parsed correctly.


**Method Detail**

### compareTo

```
public int compareTo(java.lang.Object obj)
```

:   Sorts by the sortBy field so set it before sorting.

    :   **Specified by:**: `compareTo` in interface `java.lang.Comparable`

---


### clone

```
public java.lang.Object clone()
```

:   **Overrides:**: `clone` in class `java.lang.Object`

---


### toString

```
public java.lang.String toString()
```

:   Generates a GFF3 line.

    :   **Overrides:**: `toString` in class `java.lang.Object`

---


### toStringNoAttributes

```
public java.lang.String toStringNoAttributes()
```

:   Generates a GFF3 line.

---


### sgrBundle

```
public java.lang.String sgrBundle()
```

:   Returns a collection of sgr lines representing the gff feature:
    1) seqId startMin1 0
    2) seqId start score
    3) seqId end score
    4) seqId endPlus1 0
    Does not include a final return, thus println(gff.sgrBundle()).

---


### loadAttribute

```
public static void loadAttribute(java.lang.StringBuffer sb,
                                 java.lang.String reserveWord,
                                 java.lang.String value)
```

---


### loadField

```
public static void loadField(java.lang.StringBuffer sb,
                             java.lang.String field)
```

---


### saveGFF3TextFile

```
public static void saveGFF3TextFile(Gff3Feature[] g,
                                    java.io.File newGFF3File)
```

:   Prints an array of GFF3Features as a plain text file

---


### getAlias

```
public java.lang.String getAlias()
```

:   Reserved Attribute.

---


### setAlias

```
public void setAlias(java.lang.String alias)
```

---


### getCustomAttributes

```
public java.lang.String[] getCustomAttributes()
```

:   Any Attribute that is not a reserve attribute is grouped here.
    Ie {"cat= dog", "tag=value1, value2, value3"}.

---


### setCustomAttributes

```
public void setCustomAttributes(java.lang.String[] customAttributes)
```

---


### getDbxref

```
public java.lang.String getDbxref()
```

:   Reserved Attribute.

---


### setDbxref

```
public void setDbxref(java.lang.String dbxref)
```

---


### getEnd

```
public int getEnd()
```

---


### setEnd

```
public void setEnd(int end)
```

---


### getGap

```
public java.lang.String getGap()
```

:   Reserved Attribute.

---


### setGap

```
public void setGap(java.lang.String gap)
```

---


### getId

```
public java.lang.String getId()
```

:   Reserved Attribute.

---


### setId

```
public void setId(java.lang.String id)
```

---


### getName

```
public java.lang.String getName()
```

:   Reserved Attribute.

---


### setName

```
public void setName(java.lang.String name)
```

---


### getNote

```
public java.lang.String getNote()
```

:   Reserved Attribute.

---


### setNote

```
public void setNote(java.lang.String note)
```

---


### getOntologyTerm

```
public java.lang.String getOntologyTerm()
```

:   Reserved Attribute.

---


### setOntologyTerm

```
public void setOntologyTerm(java.lang.String ontologyTerm)
```

---


### getParent

```
public java.lang.String getParent()
```

:   Reserved Attribute.

---


### setParent

```
public void setParent(java.lang.String parent)
```

---


### getPhase

```
public int getPhase()
```

---


### setPhase

```
public void setPhase(int phase)
```

---


### getScore

```
public double getScore()
```

---


### setScore

```
public void setScore(double score)
```

---


### getSeqId

```
public java.lang.String getSeqId()
```

---


### setSeqId

```
public void setSeqId(java.lang.String seqId)
```

---


### getSource

```
public java.lang.String getSource()
```

---


### setSource

```
public void setSource(java.lang.String source)
```

---


### getStart

```
public int getStart()
```

---


### setStart

```
public void setStart(int start)
```

---


### getStrand

```
public java.lang.String getStrand()
```

---


### setStrand

```
public void setStrand(java.lang.String strand)
```

---


### getTarget

```
public java.lang.String getTarget()
```

:   Reserved Attribute.

---


### setTarget

```
public void setTarget(java.lang.String target)
```

:   Reserved Attribute.

---


### getType

```
public java.lang.String getType()
```

---


### setType

```
public void setType(java.lang.String type)
```

---


### isValid

```
public boolean isValid()
```

:   Call to find out if the unparsed GFF line correctly parsed.

---


### setValid

```
public void setValid(boolean valid)
```

---


### isEndSet

```
public boolean isEndSet()
```

---


### setEndSet

```
public void setEndSet(boolean endSet)
```

---


### isPhaseSet

```
public boolean isPhaseSet()
```

---


### setPhaseSet

```
public void setPhaseSet(boolean phaseSet)
```

---


### isScoreSet

```
public boolean isScoreSet()
```

---


### setScoreSet

```
public void setScoreSet(boolean scoreSet)
```

---


### isStartSet

```
public boolean isStartSet()
```

---


### setStartSet

```
public void setStartSet(boolean startSet)
```

---


### getSortBy

```
public java.lang.String getSortBy()
```

---


### setSortBy

```
public void setSortBy(java.lang.String sortBy)
```

---


### getAttributes

```
public java.lang.String getAttributes()
```


---


|  |  |  |
| --- | --- | --- |
| **Overview** \| **Package** \| **Class** \| **Use** \| **Tree** \| **Deprecated** \| **Index** \| **Help** | |  |
| **PREV CLASS**   **NEXT CLASS** | **FRAMES**    **NO FRAMES**     **All Classes** |  |
| SUMMARY: NESTED \| FIELD \| CONSTR \| METHOD | DETAIL: FIELD \| CONSTR \| METHOD |  |


---
